# The dual architecture of digital fidelity: empirical evaluation and refinement of a theoretical model, and examination of gender invariance

**DOI:** 10.3389/fpsyg.2026.1753722

**Published:** 2026-05-22

**Authors:** Abdullah Aldemir

**Affiliations:** Department of Guidance and Psychological Counseling, Faculty of Education, Tokat Gaziosmanpaşa University, Tokat, Türkiye

**Keywords:** digital fidelity, dual architecture (theory), measurement invariance, Rasch analysis, romantic relationships

## Abstract

**Introduction:**

This study aims to empirically evaluate and refine a theoretical model that conceptualizes digital fidelity based on Individual Ethics and Relational Boundaries (The Dual Architecture).

**Methods:**

The model’s structural, discriminant, and cross-gender invariance validity were examined using both Classical Test Theory (CFA, WLS-MV) and Modern Test Theory (Rasch).

**Results:**

CFA empirically revised the two-factor Dual Architecture structure, and multi-group CFA confirmed strict invariance across genders. Nomological network analysis (relationship satisfaction, attachment styles) provided evidence for the discriminant validity of F1 and F2. Conversely, the Rasch analysis revealed a critical methodological challenge: The F1 (Digital Fidelity Ethics) sub-dimension showed unacceptably low person-separation reliability (PSI = 0.432) and a severe targeting issue (ceiling effect).

**Discussion:**

The findings indicate that the Dual Architecture model shows promising theoretical alignment and comparability; however, the Ethics dimension’s measurement using current self-report items presents significant methodological difficulties, likely due to social desirability bias.

## Introduction

Smartphones and social media have fundamentally changed the landscape of our intimate relationships (Weigel and Shrout, 2022). This digital shift has not only brought people closer but also created a great deal of uncertainty about what “fidelity” really means. The issue is no longer just about physical cheating. Online actions like “phubbing” (ignoring someone because you are on your phone), “texting” (sending secret messages), or “micro-cheating” (like liking suggestive posts) have made it hard to know what’s acceptable and what’s a betrayal (Ciarocco et al., 2012; Time Magazine, 2019; Wilson et al., 2011). This digital uncertainty can have real-world effects, as it makes people insecure, which then directly reduces satisfaction in relationships (Han et al., 2025; Ni et al., 2025). New concepts like Social Media Jealousy are now considered major dangers to the health of relationships (Krishnan et al., 2024). This creates a central conflict in modern partnerships: although we generally agree on the importance of “sexual and emotional exclusivity” (Weigel and Shrout, 2022), the way we behave shows that many of us push these boundaries to see how far we can go (Barta and Kiene, 2005; Buss and Shackelford, 1997). This highlights a critical need for holistic theoretical models to conceptualize digital fidelity and for tools to empirically test them.

### The dual architecture of digital Fidelity: individual ethics and relational boundaries

So, what makes digital fidelity so complex? Our review of the psychological literature suggests that this phenomenon is shaped by two core, conceptually distinct, yet dynamically interacting axes: Individual Fidelity Ethics and Relational Boundary Management.

1 The individual dimension: digital fidelity ethics the first dimension of fidelity is an individual struggle. It is an internal process composed of three core components:

1. The moral filter: an individual’s “ethical” judgments and “personal norms” that define what they consider to be infidelity (Lișman and Holman, 2022).
2. Self-control: the “willpower” required to apply these norms (McIntyre et al., 2015). This is the capacity to resist tempting impulses (e.g., the desire to respond to an alternative partner) that conflict with relational goals (Ciarocco et al., 2012; Fujita, 2011).
3. Transparency: the proactive behaviors of trust and digital openness that reflect this ethical stance (Doerfler et al., 2024).

Together, these three elements (Perception, Self-Control, and Transparency) form the “Digital Fidelity Ethics” structure an individual’s internalized moral compass.

2 The relational dimension: boundary management the second dimension is different. It is not about individual ethics, but about rules managed at the dyadic (couple) level. This structure is grounded in Communication Privacy Management (CPM) Theory (Petronio, 2002) and the Relationship Rules literature (Argyle and Henderson, 1984).

This dimension is not concerned with “What are *my* ethics?” but “What are *our* rules?” It is the process by which couples jointly negotiate shared boundaries (Doerfler et al., 2024) regarding what is private and what is shared in the digital space (e.g., passwords, social media interactions) (Petronio, 2002). When these negotiations fail or rules are broken, “boundary turbulence” occurs, leading to conflict and straining fidelity (McLaren and Steuber, 2012).

### Aim and contribution of the present study

Existing psychometric tools (e.g., SMJS-15, Social Media Jealousy Scale; Krishnan et al., 2024) are limited, often measuring fragmented phenomena (like jealousy or phubbing) rather than these two holistic axes (Ethics and Boundaries).

Given these theoretical gaps, the primary aim of this study is to empirically evaluate and refine the theoretical model that conceptualizes digital fidelity along this “Dual Architecture.”

This comprehensive examination investigated two key points: (1) whether this two-factor structure demonstrates discriminant validity within a nomological network (e.g., its relationship with attachment and satisfaction) and (2) whether it achieves measurement invariance across gender groups. Our attempt to test this model utilized the 10-item RRDFS form, employing both Classical Test Theory (CFA, WLS-MV) and Modern Test Theory (Rasch). This dual approach allowed us to examine not only the model’s structure but also the methodological limits of its measurability.

The core contribution of this study is twofold. First, it provides preliminary empirical support for the revised Dual Architecture model as a theoretical framework, particularly regarding its discriminant validity and cross-gender comparability. However, our second, methodological contribution is the transparent exposure of the psychometric difficulties encountered when attempting to measure this model—specifically, the ‘Individual Ethics’ dimension (e.g., low person-separation reliability and ceiling effect identified by Rasch analysis).

Therefore, this work provides a theoretical foundation, rather than a “ready-to-use scale.” It offers an empirically evaluated framework for the theory of digital fidelity and provides a clear methodological roadmap for future measurement endeavors in this area.

## Method

### Participants

To examine the scale’s structural validity, the study utilized two independent samples. Sample 1 (*N* = 285) was used for Exploratory Factor Analysis (EFA), and Sample 2 (*N* = 477) was used for Confirmatory Factor Analysis (CFA), Rasch analysis, and nomological network validation.

Detailed demographic information for both samples is provided in Supplementary Tables S1, S2. The total sample (*N* = 762) consisted primarily of women, with a mean age of 28.15 (SD = 10.03). The majority of participants were university graduates who were currently in a romantic relationship.

### Procedure

Data were collected online between September and October 2025 using a convenience sampling method. Participation was voluntary, anonymous, and based on confidentiality. After providing informed consent via an online form, participants completed the demographic questionnaire, the newly developed Digital Fidelity items, and the other validation scales. The EFA dataset (Sample 1) was collected over approximately 1 week, and the CFA dataset (Sample 2) was collected over a two-week period. After analyses for missing data and outliers, the final datasets suitable for analysis were obtained.

### Ethical approval and informed consent

The study received ethical approval from the Tokat Gaziosmanpaşa Educational Sciences Research Ethics Committee (Date: 30.10.2025, Protocol No: 01.33). Following approval, participants were recruited via an online survey. An Informed Consent Form was presented prior to participation, emphasizing that involvement was voluntary, confidential, and that participants could withdraw at any time. Only participants who provided consent were directed to the subsequent sections of the survey.

### Measures

Attachment styles questionnaire (ASQ): the short form of the Attachment Style Questionnaire (Feeney et al., 1994), adapted to 12 items by Iwanaga et al. (2018), was used to assess adult attachment styles. The Turkish adaptation and validation study was conducted by Çelik and Türk (2024). The scale consists of three subscales: “Secure Attachment,” “Anxious Attachment,” and “Avoidant Attachment.” Items are rated on a 6-point Likert scale (1 = *Strongly Disagree* to 6 = *Strongly Agree*). Higher scores on each subscale indicate a predominance of that attachment style. CFA findings from the Turkish validation (Çelik and Türk, 2024) strongly supported the three-factor structure (χ^2^/df = 2.95, CFI = 0.94, TLI = 0.92, RMSEA = 0.07, SRMR = 0.05), and convergent/discriminant validity, as well as CR and AVE values, were at expected levels.

Relationship assessment scale (RAS): the RAS (Hendrick, 1988) was used to measure relationship satisfaction. It is a 7-item, 7-point Likert-type scale. The Turkish adaptation was conducted by Curun (2001) with 140 university students. Factor analysis (Curun, 2001) confirmed a single-factor structure. The scale’s internal consistency was reported as 0.86. Two items (m4 and m7) are reverse-scored, with higher scores indicating greater relationship satisfaction.

Perceived partner’s intentions towards infidelity scale (P-ITIS): participants’ perceptions of their partner’s potential intentions to cheat were measured using the P-ITIS. The scale is based on the Turkish version (Toplu-Demirtaş and Tezer, 2013) of the Intentions Towards Infidelity Scale (ITIS; Jones et al., 2011), with the items’ perspective modified from “I” to “my partner.” Following expert opinions and cognitive interviews, an EFA confirmed a single-factor structure explaining 63.70% of the total variance. The 7-item scale uses a 7-point Likert-type rating (1 = *Not at all likely* to 7 = *extremely likely*). Higher scores indicate stronger perceptions of the partner’s intent to cheat. The Cronbach’s alpha in the Turkish adaptation study was *α* = 0.83.

Psychometric Properties of the Validation Measures (RAS, ASQ, & P-ITIS) Table 1 summarizes the CFA Fit Indices (evidence of structural validity) and internal consistency coefficients for the final measurement models of the validation scales used in this study.

**Table 1:** CFA fit indices and internal consistency coefficients for the measures (*N* = 477).

Measure/subscale	No. of Items	*χ*^2^/*df*	CFI	TLI	RMSEA	SRMR	Cronbach’s alpha (*α*)	McDonald’s omega (*ω*)
Relationship assessment scale (RAS)	5	3.14	0.999	0.999	0.067	0.007	0.94	0.95
Perceived partner’s intentions (P-ITIS)	5	11.78	0.995	0.990	0.150*	0.020	0.92	0.92
Attachment styles questionnaire (ASQ) (overall model)	10	4.54	0.968	0.953	0.086	0.050		
Secure attachment							0.76	0.78
Anxious attachment							0.58	0.58
Avoidant attachment							0.79	0.79

Table 1 displays the CFA fit indices and internal consistency coefficients for the three validation measures. The findings indicate that the scales generally possess strong psychometric properties. The 5-item single-factor model for the RAS showed excellent fit (CFI = 0.999, TLI = 0.999, RMSEA = 0.067, SRMR = 0.007) and high internal consistency (*α* = 0.94, *ω* = 0.95). The single-factor model for the P-ITIS also produced a good fit (CFI = 0.995, TLI = 0.990, SRMR = 0.020). Although the RMSEA value (0.150) should be interpreted with caution due to the low degrees of freedom (*df* = 5), the internal consistency coefficients (*α* = 0.92, *ω* = 0.92) demonstrate the scale’s sufficient measurement quality. The 10-item overall model for the ASQ provided an acceptable-to-good fit (CFI = 0.968, TLI = 0.953, RMSEA = 0.086, SRMR = 0.050). Reliability coefficients for the Secure (α = 0.76, ω = 0.78) and Avoidant (α = 0.79, ω = 0.79) subscales were satisfactory. The internal consistency of the Anxious attachment subscale was relatively low (α = 0.58, ω = 0.58), highlighting an area for future improvement for this specific subscale.

### Development of the romantic relationships digital fidelity scale (RRDFS)

The development of the Romantic Relationships Digital Fidelity Scale (RRDFS) began with a comprehensive literature review and consultation of relevant theoretical models, resulting in a large item pool of 64 items. This pool was designed to cover multiple conceptual sub-dimensions of digital fidelity (e.g., Transparency, Boundary Management, Digital Self-Regulation, Jealousy/Flirting Perception, Conflict Management, Consent for Sharing, and Repair).

To ensure content validity, experts were asked to evaluate the items using a 4-point Likert-type scale (1 = *Not Suitable*, 4 = *Very Suitable*). Based on these ratings, the Item-Content Validity Index (I-CVI) was calculated for each item, and the Scale-Content Validity Index (S-CVI/Ave) values were found to be within the 0.87–0.95 range, exceeding the accepted thresholds (≥0.80) (Waltz and Bausell, 1981; Polit and Beck, 2006).

Following content validation, cognitive interviews were conducted to assess the target audience’s comprehension of the items. Based on this feedback, items were revised for cultural appropriateness and linguistic clarity. Participants were asked if the items were clear, what they believed the items were measuring, and if they had difficulty understanding the phrasing. In response, items related to “digital transparency” and “respect for privacy” were particularly simplified, and conceptual consistency was strengthened.

After revisions, the scale was reviewed again, and a 20-item prototype form was finalized. This form was designed to test the hypothesized four-subscale structure of the RRDFS (F1: Transparency, F2: Boundary Management, F3: Flirting Perception, F4: Digital Self-Regulation) and was taken into pilot testing, forming the basis for the subsequent CFA and other analyses.

#### Data analysis

Data quality measures for EFA: to ensure data quality for the EFA (N_initial_ = 309), an Attention Check Item was included. Twenty-two participants (22) who failed this check were excluded from the analysis. Furthermore, straight-line and response variance analyses were conducted to detect uniform response patterns; participants who provided the same answer to all items or had zero variance were removed. The resulting final sample (*N* = 285) met the minimum conditions recommended for EFA (Meade and Craig, 2012; Huang et al., 2012). Descriptive statistics and normality analyses indicated that item means, standard deviations, and distribution coefficients (skewness and kurtosis) were within acceptable ranges (Ghasemi and Zahediasl, 2012; Kim, 2013). Outliers were examined using z-scores (≥ ± 3.5) and Mahalanobis distance; only 3.86% of cases exceeded the threshold. These findings confirm that assumptions were largely met, and EFA could be confidently conducted. (Detailed results are in Supplementary Table S3).

Data quality measures for CFA: prior to confirmatory factor analysis (CFA), the dataset (*N* = 477) underwent a comprehensive preliminary review. No missing data were found. No uniform response patterns (straight-lining) were detected; thus, all participants were included. Given the ordinal (Likert-type) structure of the data and indicators related to the normality assumption, the Weighted Least Squares Mean and Variance Adjusted (WLSMV) estimator was preferred over standard Maximum Likelihood (ML) (Finney and DiStefano, 2013). The fact that normality tests (e.g., Shapiro–Wilk) were significant (all *p* < 0.001), which is expected due to high sensitivity in large samples (N ≥ 300), supported this decision. Stricter thresholds for skewness (|S| ≥ 2) and kurtosis (|K| ≥ 7) were used. A high threshold (Z ≥ 3.29) was used for outlier screening, and the flagged observations were deemed to be natural reflections of the measurement, thus retaining the full dataset. (Summaries are presented in Supplementary Table S4).

EFA Prerequisite Analyses The suitability of the items for factor analysis was assessed using the Kaiser–Meyer–Olkin (KMO) measure and Bartlett’s Test of Sphericity. The KMO was 0.73, indicating the sample size was sufficient (Kaiser, 1974). Bartlett’s test was significant (*χ*^2^(190) = 1247.14, *p* < 0.001), confirming adequate correlations between items. The correlation matrix determinant was 0.011, indicating no issues with multicollinearity. The anti-image matrix diagonal values ranged from 0.58 to 0.87. These findings confirmed the dataset’s suitability for EFA. Following the removal of problematic items (m1, m2, m4, m16, and m19), the KMO remained above 0.70, and Bartlett’s test remained significant.

Determining the number of factors: the number of latent factors was determined using Horn’s (1965) parallel analysis. The eigenvalues from the actual data were compared against the 95th percentile eigenvalues derived from 1,000 random datasets generated with the same sample size and number of variables. The number of factors to retain was determined by the point at which the actual eigenvalues exceeded the random eigenvalues. As shown in Supplementary Table S11, Figure S1, the actual eigenvalues for the first four components (3.96, 2.14, 1.68, 1.39) were higher than their corresponding random eigenvalues (1.59, 1.47, 1.39, 1.33); the fifth and subsequent components fell below the random threshold. Accordingly, the scale was determined to have a four-factor structure, and subsequent CFA analyses were based on this structure.

## Results

### Exploratory factor analyses (EFA)

Following the confirmation of a four-factor structure by Parallel Analysis, an EFA was conducted on Sample 1 (*N* = 285) to conceptually refine the scale and establish its final structure. We employed Principal Axis Factoring (PAF) with a Direct Oblimin rotation, assuming inter-factor correlations.

In the first stage, the EFA results confirmed the four-factor structure. Factor loadings ranged from 0.35 to 0.83, with communalities between 0.18 and 0.70. Due to low communalities and borderline loadings, items m1, m2, and m4 were removed (*h*^2^ ≈ 0.18–0.22). For the remaining 17 items, inter-factor correlations were low (0.05–0.23). The total variance explained was 44.3% (F1 = 13.9%, F2 = 10.4%, F3 = 10.1%, F4 = 10.0%; see Supplementary Tables S5–S7).

In the second stage, after item removal, the repeated PAF + Oblimin analysis re-confirmed the four-factor structure. All items loaded above 0.30, with a range of 0.31 to 0.86, and communalities ranged from 0.32 to 0.74. Although m16 (*h*^2^ = 0.39) and m19 (*h*^2^ = 0.32) had relatively low communalities, they were retained for conceptual integrity. Inter-factor correlations remained low (0.04–0.23), and the total explained variance increased to 50.2% (F1 = 15.9%, F2 = 11.6%, F3 = 11.4%, F4 = 11.3%). These findings indicated that the four-dimensional structure was both statistically and content-wise coherent (see Supplementary Tables S8–S10).

### Final EFA analysis

The EFA conducted after further item purification confirmed that the four-factor solution was significant and interpretable. The KMO remained above 0.70, and Bartlett’s test was significant (*p* < 0.001), indicating the data was suitable for factor analysis. The four factors explained a total of 53.5% of the variance (F1 = 18.3%; F2 = 13.2%; F3 = 12.3%; F4 = 9.7%), exceeding the recommended 50% threshold for social sciences. The pattern matrix showed that item loadings ranged from 0.515 to 0.865 (all ≥0.30) and communalities ranged from 0.42 to 0.73. Acceptable, minor cross-loadings were observed only for m3 (0.37) and m10 (0.34). Inter-factor correlations were very low (*r* = 0.06–0.16), supporting the conceptual discriminant validity of the four factors (Details in Supplementary Tables S12–S14).

### Rasch analysis (modern test theory)

The rating scale model (RSM) was used to investigate unidimensionality, item fit, and category functioning based on the 4-factor EFA structure.

- Stage 1 (5-point scale): a principal component analysis (PCA) of residuals for F1, F3, and F4 showed first residual eigenvalues below 2.00, supporting unidimensionality. However, a technical convergence error occurred for F2 (its psychometric evidence was thus deferred to CFA). Infit/Outfit MNSQ values were generally within the 0.5–1.5 range, though borderline deviations were noted for F1 (m1–m6) and F3 (m11–m12). While thresholds were ordered, the MNSQ values for categories 1–3 sometimes exceeded 1.5, suggesting weak category separation.
- Stage 2 (3-point scale): to address category misfit and improve psychometric quality, all items were re-coded from 5 to 3 categories, and analyses were re-run. While model fit improved, new issues emerged: m14’s loading in F4 was weak (*λ* = 0.409), and m12 in F3 showed significant misfit, compromising the unidimensionality of that factor.
- Crucially, a CFA revealed a standardized correlation of r = 0.840 between F1 and F4, indicating severe multicollinearity and a lack of discriminant validity.

Based on this strong evidence that F1 and F4 were not distinct, a theoretical decision was made to merge F1 and F4 into a single, integrated factor. F3 (which contained the misfitting m12) was removed. Subsequent Rasch analyses were conducted on this new, theoretically revised structure (Details in Supplementary Tables S15–S22).

### Final Rasch analysis (revised two-factor model)

Based on the evidence that F1 and F4 represented a single dominant dimension (r = 0.840), the Rasch process was re-initiated assuming a two-factor structure: Factor 1 (F1 + F4 merged; 9 items) and Factor 2 (original F2; 3 items), using the 3-point scale.

This revised model proved robust:

1. Unidimensionality: the PCA of residuals for both factors showed first contrast eigenvalues below 2.00 (F1 = 1.552, F2 = 1.585), supporting the unidimensionality of each new factor.
2. Category fit: the category misfit issue was fully resolved, and item MNSQ values were stable (0.5–1.5 range).
3. DIF analysis: no statistically significant Differential Item Functioning (DIF) by gender was found for any item. The lowest *p*-value (for m15, *p* = 0.043) was well above the Bonferroni-corrected threshold (*α* = 0.0056). This provided strong evidence that the 12 items provided fair measurement for both groups.

Consequently, the Modern Test Theory (Rasch) and DIF analyses validated a two-factor, 12-item structure (F1: m1-m6, m13, m14, m15; F2: m7, m8, m9). To achieve the most parsimonious and best-fitting model for the subsequent CFA, a final decision was made to remove the two weakest items (m14 and m15) (Details in Supplementary Tables S23–S30).

### Summary of model modifications: from four factors to the dual architecture

To ensure full methodological transparency and to explicitly distinguish between theory-driven scale development decisions and *post hoc* data-driven revisions, the entire transition from the initial *a priori* four-factor model to the final two-factor model is summarized in Table 2. This summary details the sequence of item removal, category collapsing, and factor merging, alongside the specific empirical rationale or theoretical justification for each decision.

**Table 2:** Summary of scale refinement and model modifications: theory-driven vs. empirical decisions.

Refinement stage	Modification/action taken	Empirical/statistical evidence	Decision type	Total explained variance
Stage 1: EFA (Initial)	Testing the a priori 4-factor structure with the initial 20-item pool.	KMO = 0.73, Bartlett’s test *p* < 0.001. Parallel analysis suggested 4 factors (Supplementary Table S11).	Theory-driven	44.3% (Supplementary Table S7)
Stage 2: EFA (item deletion)	Removal of items m1, m2, and m4.	Low communalities (*χ*^2^ = 0.18–0.22) and borderline factor loadings (Supplementary Table S5).	Empirical	50.0% (Supplementary Table S10)
Stage 3: rasch (category collapsing)	Re-coding likert structure from 5 to 3 categories.	Severe category threshold misfit (category 1 infit MNSQ > 1.5) observed in the 5-point scale (Supplementary Table S16).	Empirical	—
Stage 4: factor merging (F1 and F4)	Merging factor 1 and factor 4 into a single “digital fidelity ethics” dimension.	Severe multicollinearity and lack of discriminant validity (*r* = 0.840 between F1 and F4). Merged factor passed unidimensionality (PCA eigenvalue <2.0).	Empirical and Theory-Driven (Data necessitated the merge, aligning with the theoretical concept of a unified internal moral compass).	—
Stage 5: factor deletion (F3)	Removal of item m12, which consequently led to the removal of Factor 3.	Item m12 showed critical Rasch misfit (Outfit MNSQ = 1.530, *t* = 5.076). removing m12 left F3 structurally weak (only 2 items).	Empirical	—
Stage 6: CFA (final item deletion)	Removal of items m14 and m15.	In the 12-item CFA, m14 showed an unacceptably weak loading (*λ* = 0.407). Subsequent 11-item CFA showed m15 had a borderline loading (*λ* = 0.511) and high SRMR (0.095).	Empirical (pursuit of a parsimonious model)	

As detailed in Table 2, the evolution of the RRDFS from its initial four-factor conceptualization to the final Dual Architecture model was governed by an iterative sequence of empirical diagnostics and theoretical recalibrations. The early stages of refinement (Stages 1 and 2) were strictly data-driven, prioritizing the elimination of indicators with insufficient shared variance to establish baseline structural coherence. However, the most substantive modification—the transition from four distinct dimensions to the consolidated two-factor model—occurred at the intersection of Modern Test Theory diagnostics and theoretical reflection (Stages 3 and 4). While the a priori model hypothesized distinct behavioral dimensions (such as self-regulation and transparency), subsequent CFA and Rasch diagnostics revealed severe multicollinearity among these facets (*r* = 0.840). This empirical redundancy necessitated a critical conceptual pivot: the data strongly indicated that these theoretically distinct behaviors were, in practice, manifestations of a singular, overarching construct. Consequently, these elements were theoretically merged into the integrated “Digital Fidelity Ethics” (F1) dimension, representing a unified internal moral compass. The subsequent removal of the misfitting third factor and the final trimming of suboptimal indicators (Stages 5 and 6) were e^x^ecuted strictly to maximize model parsimony. Ultimately, this transparent decision pathway clarifies that the final two-factor architecture is not a mere post hoc statistical artifact, but an empirically refined and theoretically coherent framework.

### Final model confirmation (CFA) (m14 and m15 removed)

Following the removal of m15 (after m14 had already been removed) due to its borderline loading and persistent model fit issues (SRMR = 0.095) in the 11-item intermediate model, the final 10-item, two-factor model was tested on the ordinal data (*N* = 477) using the WLSMV estimator. The model fit was found to be excellent (see Table 2).

As shown in Table 3, the final 10-item, two-factor model, tested with WLSMV on ordinal data, demonstrated a good fit (*χ*^2^/df = 1.76, *p* = 0.004; CFI = 0.972; TLI = 0.963; RMSEA = 0.040; SRMR = 0.080). While the *χ*^2^ was expectedly significant in a large sample, the CFI and TLI values are excellent, the RMSEA indicates a low error rate, and the SRMR is at the acceptable threshold. These findings support the structural validity of the final model.

**Table 3:** Confirmatory factor analysis fit indices for the final two-factor model (WLSMV estimator; *N* = 477).

Model fit index	Observed value	Acceptable threshold (Hu and Bentler, 1999)
*χ*^2^/*df*	1.76 (59.780/34.000)	<3.0
*p*-value	0.004	>0.05
CFI	0.972	>0.95
TLI	0.963	>0.95
RMSEA	0.040	<0.06
SRMR	0.080	≤0.08

As detailed in Table 4, the standardized factor loadings for the final model range from 0.594 to 0.905, and all are highly significant (*p* < 0.001). This pattern demonstrates that the items strongly predict their respective factors, supporting convergent validity. The inter-factor correlation is moderate (*r* = 0.444), providing strong evidence of discriminant validity and confirming that the two factors remain conceptually distinct.

**Table 4:** Standardized factor loadings and inter-factor correlation for the final 10-item, two-factor model.

Factor	Item	Standardized factor loading (std.all)
Digital fidelity ethics (7 items)	m1	0.737
	m2	0.863
	m3	0.755
	m4	0.771
	m5	0.804
	m6	0.870
	m13	0.594
Boundary management (3 items)	m7	0.703
	m8	0.905
	m9	0.680
Factor correlation		Standardized correlation (*r*)
Factor 1~~Factor 2		0.444

All factor loadings are significant (*p* < 0.001) and range from 0.594 to 0.905. All loadings exceed the 0.50 criterion, supporting strong structural validity. The inter-factor correlation (*r* = 0.444) is moderate and falls below the *r* < 0.85 threshold, indicating that the F1 and F2 structures are statistically distinct and that discriminant validity has been achieved.

### Reliability and convergent validity (classical test theory)

Reliability and validity analyses for the final 10-item, 2-factor model were conducted within the Classical Test Theory (CTT) framework (Table 4). We assessed internal consistency using Cronbach’s Alpha, McDonald’s Omega, and Composite Reliability (CR) (target ≥0.70), along with Ordinal Alpha due to the data’s nature. Convergent validity was assessed using Average Variance Extracted (AVE) (target ≥0.50).

As shown in Table 5, the AVE values exceeded the 0.50 threshold for both factors (F1 = 0.601; F2 = 0.592), supporting convergent validity. According to CTT metrics, F1 (Digital Fidelity Ethics) demonstrated high internal consistency (*α* = 0.774, *ω* = 0.776, CR = 0.791), and its Ordinal Alpha was particularly high (0.906). For F2 (Boundary Management), the classical coefficients were borderline (*α* = 0.675, CR = 0.688); however, given its Ordinal Alpha (0.807) and the small number of items (3), this was initially deemed acceptable under CTT.

**Table 5:** Reliability and convergent validity summaries for the final 10-item, 2-factor model (*N* = 477).

Factor	No. of items	Cronbach’s alpha (*α*)	McDonald’s omega (*ω*)	Composite reliability (CR/*ω*3)	Ordinal alpha (alpha.ord)	AVE (avevar)
Digital fidelity ethics (F1)	7 items	0.774	0.776	0.791	0.906	0.601
Boundary management (F2)	3 items	*0.675*	*0.695*	*0.688*	0.807	0.592

Target reliability > 0.70, Target AVE > 0.50. Values below the 0.70 threshold are italicized.

Furthermore, to stringently assess discriminant validity as recommended in recent methodological literature, the Fornell–Larcker criterion was evaluated. The square root of the AVE for F1 (0.775) and F2 (0.769) were both substantially higher than the inter-factor correlation (*r* = 0.444). This confirms that the empirical variance shared between each construct and its indicators is significantly greater than the variance shared between the two distinct constructs, providing robust evidence for discriminant validity.

While these CTT results *appear* to support the model, they must be interpreted alongside the more rigorous Modern Test Theory (Rasch) analysis, which provides a critical and contrasting picture of the scale’s reliability.

### Reliability analysis (Rasch - PSI): the critical methodological finding

In addition to CTT, reliability (internal consistency) was assessed using Modern Test Theory (MTM) via Rasch Person-Separation Reliability (PSI). This is where the critical methodological finding of the study emerged.

As shown in Table 6, the results from CTT and MTM are in stark conflict. While CTT (Ordinal Alpha = 0.906) suggested excellent reliability for F1, the Rasch PSI value was 0.432, which is considered unacceptably low and well below the 0.70 threshold. This PSI value indicates that the F1 factor, as measured, lacks the ability to reliably distinguish or “separate” individuals with high levels of “Digital Ethics” from those with low levels.

**Table 6:** Comparison of rasch reliability (PSI/EAP) with CTT Reliability (ordinal alpha) (*N* = 477).

Final factor	No. of items	Rasch reliability (PSI/EAP reliability)	CTT reliability (ordinal alpha)
Digital fidelity ethics (F1)	7 items	0.432	0.906
Boundary management (F2)	3 items	0.654	0.807

Target PSI ≥ 0.70. Values calculated on the final 10-item (7 + 3) structure.

This discrepancy suggests the high CTT alpha for F1 is a statistical artifact, likely resulting from item redundancy and the severe targeting problem (ceiling effect) identified in the Rasch analysis. As noted in the Supplementary Table S31, the targeting mismatch for F1 was +2.053 logit, confirming that the items were far too “easy” for the sample. This severe ceiling effect is the likely cause of the poor person-separation (PSI), as the scale could not differentiate among the high-scoring participants.

### Measurement invariance (MI)

To test whether the theoretical “Dual Architecture” model operates fairly and equally across gender groups (Women and Men), Measurement Invariance (MI) analyses were conducted. Analyses were performed on the final 10-item, 2-factor model using a four-step hierarchy in multi-group CFA (with WLSMV estimator) appropriate for 3-point ordinal data.

As seen in Table 7, the final 10-item, 2-factor model achieved full and strict invariance across genders.

1. Configural: the baseline model fit was high (CFI = 0.963, RMSEA = 0.038), showing the factor structure is valid for both groups.
2. Metric: constraining the loadings (Metric) resulted in changes (ΔCFI = 0.006; ΔRMSEA = 0.008) well within acceptable limits, indicating the items carry the same meaning for men and women.
3. Scalar: constraining the thresholds (Scalar) also passed (ΔCFI = 0.004; ΔRMSEA = −0.002), allowing for the valid comparison of latent means.
4. Strict: even the strict model (constraining error variances) passed (ΔCFI = 0.003; ΔRMSEA = 0.002).

**Table 7:** Measurement invariance test results for the final 10-item model (group: gender).

Invariance stage	Model compared	CFI	TLI	RMSEA	ΔCFI	ΔRMSEA
1. Configural	Baseline model (groups free)	0.963	0.953	0.038	—	—
2. Metric	Loadings constrained	0.957	0.952	0.046	0.006	0.008
3. Scalar	Loadings + thresholds constrained	0.961	0.960	0.044	0.004	−0.002
4. Strict	Loadings + thresholds + residuals constrained	0.958	0.957	0.046	0.003	0.002

Criteria applied: ΔCFI < 0.010 and ΔRMSEA < 0.015. All models maintained CFI/TLI > 0.95 and RMSEA < 0.06.

This is a key theoretical finding: it confirms that the “Dual Architecture” construct (the theory) is understood and structured identically by both men and women, even if the measurement of F1 is flawed (as shown by Rasch).

### Psychometric properties of validation measures

CFA fit indices and internal consistency coefficients for the external criteria measures (RAS, ASQ, and P-ITIS) were summarized in Table 1 (Method section). Further details supporting their structural validity are provided below and in the Supplementary Tables S32–S36.

Detailed Psychometric Evidence All items in the final models for the single-factor RAS and P-ITIS, and the three-factor ASQ, were significant (*p* < 0.001). Standardized factor loadings and explained variances (*R*^2^) were high, supporting their convergent validity.

Discriminant Validity (ASQ) Inter-factor correlations in the multi-dimensional ASQ were examined. The highest correlation (*r* = 0.723) remained below the threshold, indicating the factors are statistically distinct and supporting the scale’s discriminant validity (see Supplementary Table S35).

### Criterion and nomological network validity

To support the construct validity of the RRDFS and test its nomological network, we examined the relationships between its sub-factors and external criteria (Perceived Partner’s Infidelity Intentions, Relationship Satisfaction, and Attachment Styles) using Pearson Correlation analysis (Table 8).

**Table 8:** Pearson correlations between subscales and criterion variables (*N* = 477; two-tailed).

Variable	1	2	3	4	5	6	7	8	9
1. Digital fidelity ethics (F1)	—								
2. Boundary management (F2)	0.31*	—							
3. RRDFS_TOTAL	0.87*	0.75*	—						
4. Partner’s infidelity intentions	−0.07	−0.09	−0.09*	—					
5. Relationship satisfaction	0.11*	0.13*	0.15*	−0.40*	—				
6. Secure attachment	−0.17*	0.02	−0.13*	0.02	−0.03	—			
7. Anxious attachment	0.03	0.16*	0.10*	0.23*	−0.19*	0.16*	—		
8. Avoidant attachment	0.07	0.11*	0.10*	0.12*	−0.10*	−0.31*	0.49*	—	
9. Attachment_total	−0.03	0.10*	0.03	0.14*	−0.13*	−0.70*	0.65*	0.85*	—

**p < 0.05. *p*-values adjusted using Holm-Bonferroni correction. Secure attachment and attachment total are reverse-coded.

As shown in Table 8, the F1 and F2 sub-factors are moderately correlated (*r* ≈ 0.31), and both correlate highly with the total score (*r* = 0.87 and *r* = 0.75, respectively). Small, consistent positive correlations were observed with Relationship Satisfaction (*r* ≈ 0.11–0.15). Relationships with Partner’s Infidelity Intentions were weak/negative. Regarding attachment, Secure Attachment (reverse-coded) correlated negatively with RRDFS (*r* = −0.13 to −0.17), while small-to-moderate positive correlations were found with the other insecure attachment styles (*r* ≈ 0.10–0.23).

### Establishing the nomological network (discriminant validity)

To test the external validity of the “Dual Architecture” and, critically, to establish the discriminant validity of the two factors, we examined the extent to which F1 and F2 were *independently* predicted by established criteria (Partner’s Infidelity Intentions, Relationship Satisfaction, and Attachment Styles) using Multiple Linear Regression (MLR). All continuous variables were standardized.

The regression model predicting Digital Fidelity Ethics (F1) (Table 9) was significant (*R*^2^ = 0.078, *p* < 0.001). The significant positive predictors for F1 were Relationship Satisfaction (*β* = 0.126) and Avoidant Attachment (*β* = 0.129). Secure Attachment (*β* = −0.202) and Male Gender (*β* = −0.190) were negative predictors. Crucially, Anxious Attachment was not a significant predictor of F1 (*p* = 0.819).

**Table 9:** Multiple linear regression predicting digital fidelity ethics (F1) (standardized coefficients; *N* = 477).

Predictor variable	*β*	SE	*t*	*p*
(Constant)	0.093	0.064	1.469	0.143
Partner’s infidelity intentions	−0.059	0.050	−1.196	0.232
Relationship satisfaction	0.126	0.049	2.553	0.011
Secure attachment	−0.202	0.047	−4.307	< 0.001
Anxious attachment	0.012	0.052	0.229	0.819
Avoidant attachment	0.129	0.053	2.453	0.015
Age	−0.073	0.047	−1.559	0.120
Gender_2 (male)	−0.190	0.093	−2.048	0.041
Model summary	*R*^2^ = 0.078		F(8, 468) = 5.702	*p* < 0.001

The model predicting boundary management (F2) (Table 10) was also significant (*R*^2^ = 0.074, *p* < 0.001). Significant predictors were Relationship Satisfaction (*β* = 0.136) and Male Gender (*β* = 0.238). Most importantly, Anxious Attachment was a strong positive predictor (*β* = 0.184, *p* < 0.001). Notably, Secure and Avoidant Attachment were not significant predictors of F2 (*p* > 0.05).

**Table 10:** Multiple linear regression predicting boundary management (F2) (standardized coefficients; *N* = 477).

Predictor variable	*β*	SE	*t*	*p*
(Constant)	−0.117	0.064	−1.829	0.068
Partner’s infidelity intentions	−0.062	0.050	−1.249	0.212
Relationship satisfaction	0.136	0.050	2.741	0.006
Secure attachment	−0.063	0.047	−1.348	0.178
Anxious attachment	0.184	0.052	3.509	< 0.001
Avoidant attachment	0.063	0.053	1.198	0.231
Age	−0.044	0.047	−0.935	0.350
Gender_2 (male)	0.238	0.093	2.551	0.011
Model summary	*R*^2^ = 0.074		F(8, 468) = 5.370	*p* < 0.001

These two tables provide preliminary nomological support for the theoretical divergence of the “Dual Architecture” model. The findings demonstrate a clear psychological divergence:

- F1 (Ethics), an internal, individual process, is significantly predicted by Avoidant Attachment.
- F2 (Boundaries), a dyadic, relational process, is significantly predicted by Anxious Attachment.

## Discussion, conclusion, and suggestions

The primary aim of this study was to empirically test the validity of the theoretical model conceptualizing digital fidelity along two core axes (the “Dual Architecture”): Individual Ethics (F1) and Relational Boundaries (F2). Our comprehensive analyses (CFA, Rasch, Invariance) demonstrated that while this theoretical model is structurally robust and meaningful, the attempt to measure it via self-report particularly the F1 dimension—concurrently revealed significant methodological challenges.

### Theoretical contribution: empirical support for the revised dual architecture model

A key theoretical finding of this study is the empirical evidence suggesting that, within the parameters of self-report measurement, digital fidelity organizes in the minds of participants as an integrated “Digital Fidelity Ethics” (F1) factor, rather than the four discrete sub-dimensions originally hypothesized (e.g., transparency, self-regulation). The high correlation found during the EFA (r = 0.840 between F1 and F4) suggests that concepts like the “Moral Filter” (Lișman and Holman, 2022) and “Self-Control” (Ciarocco et al., 2012) are not *separate parts* of fidelity, but rather different manifestations of the same internal moral compass.

The second key theoretical finding is that “Boundary Management” (F2) is statistically distinct from “Digital Fidelity Ethics” (F1), as evidenced by their moderate correlation (r = 0.444). This distinction strongly supports the proposed theoretical architecture: F1 is an *intra-individual* process (ethics), while F2 is a *dyadic* process (rules). This finding aligns perfectly with established relational theories, particularly Petronio’s (2002) Communication Privacy Management (CPM) Theory and the “Relationship Rules” approach (Argyle and Henderson, 1984).

The theoretical robustness of this model was corroborated not just structurally (via CFA fit indices; see Table 2), but more importantly, through its nomological network and measurement invariance analyses:

1. Discriminant validity (the nomological network): Although the explained variance is relatively modest, the regression analyses (Tables 8, 9) offered valuable preliminary evidence of the model’s theoretical coherence. F1 and F2 were predicted by different psychological antecedents. F1 (Ethics), an internal process, was significantly predicted by Avoidant Attachment. In contrast, F2 (Boundaries), a dyadic and relational process, was significantly predicted by Anxious Attachment. This confirms that the two factors are not merely a statistical artifact but possess distinct psychological underpinnings.
2. Comparability (measurement invariance): one of the study’s strongest contributions is the evidence of Scalar and Strict Invariance across genders (ΔCFI ≤ 0.006). This is critical evidence that legitimizes gender comparisons in the field, demonstrating that the “Dual Architecture” *theory* holds the same meaning and structure for both men and women, allowing for valid (latent mean) comparisons.

This theoretical divergence also offers a clear conceptual roadmap for interventions. Problems stemming from F1 (Ethics) may require an individual focus (on moral reasoning or self-control), whereas problems with F2 (Boundaries) necessitate a relational, dyadic focus (on CPM-based negotiation) (Lișman and Holman, 2022; Petronio, 2002).

### Methodological contribution: the challenge of measuring individual ethics

While this study validated the theoretical model, it also transparently exposed the psychometric weaknesses of the measurement attempt (the RRDFS-10). These weaknesses should not be interpreted merely as a “flawed scale,” but rather as empirical evidence of a “hard-to-measure construct.”

The core methodological finding is the failure in measuring the F1 (Digital Fidelity Ethics) dimension. While Classical Test Theory (CTT) suggested high reliability (Ordinal Alpha = 0.906), Modern Test Theory (Rasch) revealed this finding to be a statistical illusion.

The Rasch analysis demonstrated unacceptably low person-separation reliability (PSI = 0.432) and a severe targeting problem. This indicates that the F1 items were “too easy” for the sample, creating a significant ceiling effect.

A plausible hypothesis for this methodological finding is the influence of social desirability bias (SDB). On a topic as sensitive as “fidelity,” participants are highly unlikely to use a self-report measure to label themselves as “unethical.” Consequently, the low PSI for F1 is not merely a flaw of this specific scale, but rather empirical evidence that measuring a construct like “digital ethics”—which is highly susceptible to SDB—is nearly impossible using traditional self-report methods.

### Limitations and future directions

Beyond the core methodological findings (PSI, SDB) discussed above, the study has several standard limitations. The use of convenience sampling limits generalizability, and the lack of test–retest reliability data means we cannot speak to the scale’s temporal stability.

A specific consideration regards the Boundary Management (F2) dimension, which comprises only three items. While the exceptionally high factor loadings (reaching 0.928) suggest these items effectively capture a core statistical aspect of the construct, we must explicitly acknowledge that representing a complex relational dimension like boundary management with only three indicators fundamentally limits its content coverage. The borderline classical reliability (CR = 0.688) further underscores this limitation. Therefore, while these three items serve as a functional preliminary proxy in the current study, future research must prioritize developing and adding complementary items to adequately capture the full conceptual breadth and complexity of dyadic digital boundary negotiations.

A further methodological limitation pertains to the external validation measures utilized in this study. Specifically, the Perceived Partner’s Intentions Towards Infidelity Scale (P-ITIS) yielded an elevated RMSEA value (0.150)—likely exacerbated by its low degrees of freedom (df = 5)—and the anxious attachment subscale demonstrated relatively low internal consistency (*α* = 0.58). While these values do not invalidate the preliminary nomological network established herein, they inherently introduce measurement error into the regression models. Consequently, the structural relationships observed between digital fidelity dimensions and these specific external criteria should be interpreted with appropriate caution, and future research should consider employing supplementary or more robust validators to replicate these nomological findings.

Based on these findings, future research should proceed on two tracks:

1. Use of the theoretical model: the “Dual Architecture” model, empirically supported here (via discriminant validity and gender invariance), can be confidently used as a theoretical framework in future longitudinal or causal studies examining digital fidelity (e.g., its relationship with the Dark Triad or relationship quality).
2. Improvement of measurement: future scale revisions must solve two distinct problems: (a) new items must be added to F2 (Boundary Management) to improve its content coverage and reliability; and (b) addressing the measurement of Digital Fidelity Ethics (F1) necessitates a methodological shift beyond traditional self-report. Given the confirmed ceiling effect and social desirability bias, future research should prioritize indirect measurement strategies—such as vignette-based situational judgment tests or implicit association tests (IAT). These approaches are likely to be more effective in bypassing the conscious moral filtering of participants, thereby capturing the true variance in individual ethical stances.

## Conclusion

This study offers a significant theoretical contribution by conceptualizing digital fidelity via the “Dual Architecture” model. The primary contribution of this work to the literature is not to offer a “finished” scale, but to empirically highlight two key insights:

1. Theoretical contribution: the revised “Dual Architecture” of digital fidelity, comprising “Individual Ethics” and “Relational Boundaries,” shows promising structural coherence and cross-gender comparability, though its full theoretical potential requires alternative measurement approaches.
2. Methodological contribution: measuring the “Individual Ethics” dimension with high precision does not yet seem possible using traditional self-report methods, as demonstrated by the critically low PSI (0.432) and the severe ceiling effect.

## Data Availability

The datasets presented in this study can be found in online repositories. The names of the repository/repositories and accession number(s) can be found below: Harvard Dataverse/https://doi.org/10.7910/DVN/TIEJRX.
