# Supplementary material for: The dual architecture of digital fidelity: empirical evaluation and refinement of a theoretical model, and examination of gender invariance
Source: Front Psychol. 2026 May 22;17:1753722. doi: 10.3389/fpsyg.2026.1753722 (PMC13236677; doi:10.3389/fpsyg.2026.1753722)
Supplement: Supplementary file 1 [file Table_1.docx]

**SUPPLEMENTARY FILE**

**Participants and Samples (EFA, CFA)**

Two independent samples were used in this study to test structural validity. Sample 1 (N = 285) was used for Exploratory Factor Analysis (EFA). Sample 2 (N = 477) was used for Confirmatory Factor Analysis (CFA), criterion validity, and Rasch analyses. Demographic distributions for both datasets are presented in Table S1 and Table S2, respectively.

Table S1. Demographic characteristics of participants (Sample 1; EFA; N = 285).

| Variable | Category | f | % |
| --- | --- | --- | --- |
| Gender | Female | 183 | 64.2 |
|  | Male | 102 | 35.8 |
| Age Range | 18-23 | 160 | 56.1 |
|  | 24-29 | 24 | 8.4 |
|  | 30-35 | 37 | 13.0 |
|  | 36-40 | 37 | 13.0 |
|  | 41-45 | 27 | 9.5 |
| Education Level | High School | 53 | 18.6 |
|  | Associate Degree | 22 | 7.7 |
|  | Bachelor's Degree | 190 | 66.7 |
|  | Master's Degree | 18 | 6.3 |
|  | Doctorate | 2 | 0.7 |
| Relationship Status | Single | 115 | 40.4 |
|  | In a relationship | 66 | 23.2 |
|  | Married | 96 | 33.7 |
|  | Separated | 7 | 2.5 |
|  | Engaged | 1 | 0.4 |
| Living Situation | Living together | 110 | 38.6 |
|  | Living separately | 175 | 61.4 |
| Relationship Duration | 1-12 months | 143 | 50.2 |
|  | 1-5 years | 59 | 20.7 |
|  | 6-10 years | 23 | 8.1 |
|  | 11-15 years | 30 | 10.5 |
|  | 16-20 years | 19 | 6.7 |
|  | 21-25 years | 11 | 3.9 |
| Daily Social Media Use | 0-30 min | 5 | 1.8 |
|  | 31-60 min | 23 | 8.1 |
|  | 1-2 hours | 112 | 39.3 |
|  | 2-4 hours | 98 | 34.4 |
|  | 4+ hours | 47 | 16.5 |
| Total |  | 285 | 100 |

The first dataset (Sample 1), collected via convenience sampling for Exploratory Factor Analysis (EFA), consisted of N = 285 participants. Distributions of demographic and study-specific variables for Sample 1 are provided in Table S1. Women constituted 64.2% (n = 183) of the sample, and men constituted 35.8% (n = 102). The majority of participants were in the 18–23 age range (56.1%; n = 160) and held a bachelor's degree (66.7%; n = 190). Regarding relationship status, 40.4% (n = 115) reported being single, and 61.4% (n = 175) reported not living in the same household as their partner.

Table S2. Demographic characteristics of participants (Sample 2; CFA and additional analyses; N = 477).

| Variable | Category | f | % |
| --- | --- | --- | --- |
| Gender | Female | 243 | 50.9 |
|  | Male | 234 | 49.1 |
| Age Range | 18-23 | 251 | 52.6 |
|  | 24-29 | 30 | 6.3 |
|  | 30-35 | 72 | 15.1 |
|  | 36-40 | 51 | 10.7 |
|  | 41-45 | 73 | 15.3 |
| Relationship Duration | 1-11 months | 213 | 44.7 |
|  | 1-5 years | 145 | 30.4 |
|  | 6-10 years | 36 | 7.5 |
|  | 11-15 years | 23 | 4.8 |
|  | 16-20 years | 27 | 5.7 |
|  | 21-25 years | 33 | 6.9 |
| Relationship Preference | Monogamous | 437 | 91.6 |
|  | Open relationship | 27 | 5.7 |
|  | Undecided | 13 | 2.7 |
| Total |  | 477 | 100.0 |

The second dataset (Sample 2), used for Confirmatory Factor Analysis (CFA), criterion validity, Rasch analysis, and advanced analyses, comprised N = 477 participants. Detailed distributions for Sample 2 are presented in Table S2. The gender distribution in this sample was approximately balanced, with women accounting for 50.9% (n = 243) and men 49.1% (n = 234). The mean age of participants was 28.15 (SD = 10.03). In terms of relationship preference, 91.6% (n = 437) of the sample described their relationships as monogamous.

**Z-Score and Normality Analysis for EFA**

Table S3. Descriptive statistics and normality test results for EFA items (Sample 1; N = 285).

| Item | N | Mean | SD | Skewness | Kurtosis | Shapiro-Wilk p | D’Agostino K² | K² p | \|z\|>3 (%) |
| --- | --- | --- | --- | --- | --- | --- | --- | --- | --- |
| m1 | 285 | 3.92 | 0.91 | −1.14 | 0.43 | < .001 | 56.78 | < .001 | 2.81 |
| m2 | 285 | 4.40 | 0.66 | −1.33 | 0.58 | < .001 | 86.55 | < .001 | 1.05 |
| m3 | 285 | 4.48 | 0.80 | −1.98 | 3.01 | < .001 | 126.22 | < .001 | 3.51 |
| m4 | 285 | 3.61 | 1.02 | −0.68 | −0.27 | < .001 | 19.18 | < .001 | 0.00 |
| m5 | 285 | 3.86 | 1.08 | −0.92 | −0.24 | < .001 | 31.92 | < .001 | 0.00 |
| m6 | 285 | 2.72 | 1.09 | 0.40 | −0.70 | < .001 | 24.77 | < .001 | 0.00 |
| m7 | 285 | 3.74 | 1.03 | −0.88 | −0.33 | < .001 | 30.19 | < .001 | 0.00 |
| m8 | 285 | 3.40 | 1.16 | −0.38 | −0.89 | < .001 | 47.46 | < .001 | 0.00 |
| m9 | 285 | 3.95 | 1.03 | −1.07 | 0.13 | < .001 | 43.73 | < .001 | 0.00 |
| m10 | 285 | 3.89 | 1.02 | −1.07 | 0.16 | < .001 | 43.30 | < .001 | 0.00 |
| m11 | 285 | 4.49 | 0.78 | −1.95 | 2.23 | < .001 | 124.48 | < .001 | 3.51 |
| m12 | 285 | 4.73 | 0.55 | −1.90 | 2.02 | < .001 | 141.88 | < .001 | 3.86 |
| m13 | 285 | 4.62 | 0.62 | −1.87 | 2.20 | < .001 | 121.03 | < .001 | 0.70 |
| m14 | 285 | 4.55 | 0.75 | −1.94 | 2.15 | < .001 | 120.22 | < .001 | 3.51 |
| m15 | 285 | 4.76 | 0.57 | −1.33 | 1.14 | < .001 | 214.40 | < .001 | 3.16 |
| m16 | 285 | 3.93 | 1.00 | −0.93 | −0.25 | < .001 | 33.49 | < .001 | 0.00 |
| m17 | 285 | 4.50 | 0.61 | −1.29 | 0.86 | < .001 | 79.08 | < .001 | 0.70 |
| m18 | 285 | 3.69 | 1.08 | −0.58 | −0.37 | < .001 | 18.09 | < .001 | 0.00 |
| m19 | 285 | 4.50 | 0.61 | −1.16 | 0.44 | < .001 | 52.89 | < .001 | 1.05 |
| m20 | 285 | 3.91 | 0.99 | −0.83 | −0.19 | < .001 | 28.19 | < .001 | 0.00 |
| Note. SD = Standard Deviation; all tests are reported as two-tailed. The \|z\|>3 ratio indicates the percentage of univariate outliers. | | | | | | | | | |

Descriptive statistics and normality analyses for the scale items are presented in Table S3. Item means (2.72 to 4.76) and standard deviations (0.55 to 1.16) indicated sufficient variance and response homogeneity. Skewness (−1.98 to 0.40) and kurtosis (−0.89 to 3.01) values were generally within acceptable ranges (Ghasemi & Zahediasl, 2012; Kim, 2013). Although the Shapiro–Wilk and D’Agostino K² tests were significant—a common occurrence due to high sensitivity in large samples, especially with Likert-type data—the normality assumption was considered practically met. In the outlier analysis, using a |z| > 3 criterion, no item exceeded a 5% outlier rate (highest was 3.86%). Therefore, we concluded that no significant univariate outliers were present and that all items were suitable for inclusion in the EFA.

**Z-Score and Normality Analysis for CFA and Other Analyses**

Table S4. Descriptive statistics and detailed normality analysis results for CFA and additional analyses (N = 477).

| Item | Mean | SD | Skewness | Kurtosis | Shapiro-Wilk | p (S-W) | D'Agostino K2 | p (D'A) | \|z\|>3 (%) |
| --- | --- | --- | --- | --- | --- | --- | --- | --- | --- |
| m1 | 4.409 | 0.902 | -1.968 | 4.105 | 0.664 | <.001 | 195.223 | <.001 | 2.52 |
| m2 | 4.505 | 0.878 | -2.444 | 6.482 | 0.588 | <.001 | 255.771 | <.001 | 3.14 |
| m3 | 4.595 | 0.752 | -2.400 | 6.796 | 0.584 | <.001 | 254.604 | <.001 | 2.73 |
| m4 | 4.438 | 0.854 | -1.867 | 3.803 | 0.674 | <.001 | 183.349 | <.001 | 1.68 |
| m5 | 4.497 | 0.827 | -2.175 | 5.554 | 0.633 | <.001 | 226.379 | <.001 | 3.35 |
| m6 | 4.730 | 0.692 | -3.399 | 13.120 | 0.441 | <.001 | 364.035 | <.001 | 2.52 |
| m7 | 2.958 | 1.296 | 0.229 | -1.178 | 0.884 | <.001 | 261.015 | <.001 | 0.00 |
| m8 | 3.740 | 1.098 | -0.705 | -0.320 | 0.860 | <.001 | 36.047 | <.001 | 0.00 |
| m9 | 3.329 | 1.263 | -0.274 | -1.086 | 0.891 | <.001 | 154.753 | <.001 | 0.00 |
| m10 | 3.774 | 1.240 | -0.905 | -0.247 | 0.821 | <.001 | 51.587 | <.001 | 0.00 |
| m11 | 3.746 | 1.190 | -0.817 | -0.313 | 0.839 | <.001 | 45.131 | <.001 | 0.00 |
| m12 | 3.943 | 1.055 | -1.074 | 0.615 | 0.814 | <.001 | 70.352 | <.001 | 0.00 |
| m13 | 4.581 | 0.701 | -2.179 | 6.254 | 0.618 | <.001 | 232.510 | <.001 | 2.10 |
| m14 | 3.755 | 1.162 | -0.707 | -0.474 | 0.854 | <.001 | 40.997 | <.001 | 0.00 |
| m15 | 3.782 | 1.072 | -0.809 | 0.225 | 0.859 | <.001 | 43.070 | <.001 | 0.00 |
| k1 | 5.119 | 2.134 | -0.804 | -0.700 | 0.881 | <.001 | 65.228 | <.001 | 0.00 |
| k2 | 5.222 | 2.098 | -0.901 | -0.514 | 0.790 | <.001 | 59.107 | <.001 | 0.00 |
| k3 | 5.287 | 2.033 | -0.944 | -0.391 | 0.793 | <.001 | 57.991 | <.001 | 0.00 |
| k4 | 2.358 | 1.913 | 1.330 | 0.564 | 0.726 | <.001 | 92.886 | <.001 | 0.00 |
| k5 | 4.866 | 2.049 | -0.568 | -0.892 | 0.861 | <.001 | 80.500 | <.001 | 0.00 |
| k6 | 5.715 | 2.003 | -1.391 | 0.512 | 0.673 | <.001 | 97.702 | <.001 | 0.00 |
| k7 | 3.094 | 1.936 | 0.741 | -0.519 | 0.869 | <.001 | 45.877 | <.001 | 0.00 |
| n1 | 4.067 | 1.405 | -0.767 | -0.000 | 0.877 | <.001 | 38.513 | <.001 | 0.00 |
| n2 | 3.943 | 1.378 | -0.498 | -0.409 | 0.909 | <.001 | 23.163 | <.001 | 0.00 |
| n3 | 4.541 | 1.262 | -1.005 | 0.865 | 0.862 | <.001 | 67.624 | <.001 | 0.00 |
| n4 | 4.556 | 1.168 | -1.095 | 1.333 | 0.853 | <.001 | 82.460 | <.001 | 3.35 |
| n5 | 2.883 | 1.353 | 0.276 | -0.720 | 0.916 | <.001 | 31.948 | <.001 | 0.00 |
| n6 | 3.421 | 1.549 | -0.092 | -0.987 | 0.917 | <.001 | 90.466 | <.001 | 0.00 |
| n7 | 2.945 | 1.541 | 0.299 | -1.000 | 0.903 | <.001 | 102.921 | <.001 | 0.00 |
| n8 | 2.463 | 1.473 | 0.807 | -0.344 | 0.853 | <.001 | 44.965 | <.001 | 0.00 |
| n9 | 3.375 | 1.519 | -0.001 | -0.911 | 0.920 | <.001 | 62.584 | <.001 | 0.00 |
| n10 | 3.969 | 1.421 | -0.479 | -0.487 | 0.914 | <.001 | 24.911 | <.001 | 0.00 |
| n11 | 3.719 | 1.429 | -0.338 | -0.659 | 0.925 | <.001 | 28.312 | <.001 | 0.00 |
| n12 | 3.327 | 1.528 | 0.052 | -0.992 | 0.925 | <.001 | 92.471 | <.001 | 0.00 |
| h1 | 2.149 | 1.930 | 1.627 | 1.275 | 0.637 | <.001 | 129.475 | <.001 | 0.00 |
| h2 | 2.277 | 2.029 | 1.449 | 0.622 | 0.660 | <.001 | 104.326 | <.001 | 0.00 |
| h3 | 2.166 | 1.905 | 1.579 | 1.163 | 0.653 | <.001 | 123.549 | <.001 | 0.00 |
| h4 | 2.184 | 1.867 | 1.511 | 1.010 | 0.675 | <.001 | 115.219 | <.001 | 0.00 |
| h5 | 2.178 | 1.886 | 1.578 | 1.205 | 0.664 | <.001 | 124.078 | <.001 | 0.00 |
| Note: N = 477. SD = Standard Deviation. Skewness and Kurtosis values are presented for the assessment of the normality assumption. The \|z\|>3 (%) column indicates the percentage of outliers exceeding the \|z\| > 3 criterion for each item. M1-M15 = RRDFS item, k1-k7 = RAS item, n1-n12 = ASQ item, h1-h5 = P-ITIS item | | | | | | | | | |

Descriptive statistics and detailed normality analyses for the items are presented in Table S4. Item means ranged from 2.35 to 5.22, and standard deviations from 0.87 to 2.13. Skewness values were between −2.40 and 1.33, and kurtosis values between −0.89 and 13.12. Given that all Shapiro–Wilk and D’Agostino K² tests were significant (*p* < .001), which is expected in large samples, the normality assessment was based on critical skewness and kurtosis coefficients. Significant skewness (|S| > 2) was observed for five items, and significant kurtosis (|K| > 7) was observed for one item (m6, S = −3.40, K = 13.12). In the outlier analysis (|z| > 3), 80 individual data points were flagged. These were assessed to reflect the natural variability of the sample rather than data entry errors and were thus retained. Overall, the normality assumption was considered practically met, and all items were deemed appropriate for inclusion in the subsequent analyses.

**EFA ANALYSES**

**EFA Stage 1 (All items m1–m20)**

Table S5. Exploratory factor analysis results: pattern loadings, communalities (h²), and factor structure (m1–m20).

| Item | F1 | F2 | F3 | F4 | h² |
| --- | --- | --- | --- | --- | --- |
| m1 |  |  | .35 |  | .18 |
| m2 |  |  |  |  | .22 |
| m3 | .52 | .39 |  |  | .47 |
| m4 |  | .43 |  |  | .22 |
| m5 |  |  | .82 |  | .68 |
| m6 |  | .67 |  |  | .47 |
| m7 |  | .78 |  |  | .61 |
| m8 |  | .62 |  |  | .43 |
| m9 |  |  | .83 |  | .70 |
| m10 |  |  | .54 |  | .41 |
| m11 | .61 |  |  |  | .39 |
| m12 | .82 |  |  |  | .67 |
| m13 | .63 |  |  |  | .48 |
| m14 | .55 |  |  |  | .37 |
| m15 | .74 |  |  |  | .55 |
| m16 |  |  |  | .56 | .33 |
| m17 |  |  |  | .68 | .50 |
| m18 |  |  |  | .64 | .43 |
| m19 |  |  |  | .48 | .34 |
| m20 |  |  |  | .49 | .40 |
| Note. Factor loadings below .30 are not shown in the table. h² = Communality (common variance). | | | | | |

Table S5 presents the EFA results for the digital fidelity scale. Factor loadings ranged from 0.35 to 0.83, indicating that items were significantly related to their respective factors. Loadings above 0.30 (Hair et al., 2021) suggest items adequately represent their factors. The majority of items loaded significantly on a single factor, with no cross-loadings (loadings > 0.30 on more than one factor) observed, indicating clear factor separation consistent with the theoretical structure. Communalities (h²) ranged from 0.18 to 0.70. While most items were above 0.40, some (e.g., m1, m2, m4, m11, m14, m16, m19) showed lower communalities (0.18–0.37). These items were retained at this stage to preserve theoretical integrity and were re-evaluated during the CFA phase. Overall, the EFA supported the four-dimensional structure.

Table S6. Inter-factor correlations (oblimin rotation).

|  | F1 | F2 | F3 | F4 |
| --- | --- | --- | --- | --- |
| F1 | 1 | .049 | .153 | .234 |
| F2 |  | 1 | .085 | .151 |
| F3 |  |  | 1 | .132 |
| F4 |  |  |  | 1 |
| Note. Inter-factor correlations are low (r = .05–.23), which indicates that the factors are conceptually distinct. | | | | |

Table S6 presents the inter-factor correlation coefficients. The values ranged from 0.05 to 0.23, indicating weak to moderate relationships. These low correlations support the use of the oblimin (oblique) rotation, aligning with the assumption that the subscales are theoretically related but not redundant (Tabachnick & Fidell, 2019). This demonstrates that structural discriminant validity was achieved, supporting the multidimensional structure.

Table S7. Total variance explained by the four-factor solution.

| Factor | SS Loadings | Variance Explained (%) | Cumulative Variance (%) |
| --- | --- | --- | --- |
| F1 | 2.78 | 13.90 | 13.90 |
| F2 | 2.07 | 10.40 | 24.30 |
| F3 | 2.01 | 10.10 | 34.30 |
| F4 | 1.99 | 10.00 | 44.30 |
| Note. The four factors explain 44.3% of the total variance. | | | |

Table S7 shows the total variance explained by the four-factor solution, which was 44.3%. The individual factors explained 13.9%, 10.4%, 10.1%, and 10.0%, respectively. This total variance is consistent with the acceptable 40%–60% range for psychological scales (Hair et al., 2021; Kline, 2023), indicating the model explains a significant portion of the variance.

**EFA Stage 2 (Items m1, m2, m4 removed)**

Table S8. Pattern matrix and communalities (h²) after removing items m1, m2, and m4.

| Item | F1 | F2 | F3 | F4 | Communality |
| --- | --- | --- | --- | --- | --- |
| m3 | .51 | .35 |  |  | .42 |
| m5 |  |  | .85 |  | .73 |
| m6 |  | .73 |  |  | .54 |
| m7 |  | .78 |  |  | .62 |
| m8 |  | .62 |  |  | .44 |
| m9 |  |  | .86 |  | .74 |
| m10 |  | .33 | .53 |  | .41 |
| m11 | .62 |  |  |  | .40 |
| m12 | .82 |  |  |  | .68 |
| m13 | .62 |  | .31 |  | .49 |
| m14 | .55 |  |  |  | .40 |
| m15 | .76 |  |  |  | .59 |
| m16 |  |  |  | .61 | .39 |
| m17 |  |  |  | .69 | .51 |
| m18 |  |  |  | .64 | .43 |
| m19 |  |  |  | .44 | .32 |
| m20 |  | .35 |  | .50 | .40 |
| **Note.** Only factor loadings ≥ 0.30 are reported. Extraction method: Principal Axis Factoring (PAF); rotation: Direct Oblimin. | | | | | |

The pattern loadings in Table S8 show that the four-factor structure remains statistically strong and interpretable. All items have factor loadings above 0.30. Factor 1 (m3, m11-m15) is the dominant factor (loadings .51–.82). Factors 2 (m6-m8), 3 (m5, m9, m10), and 4 (m16-m20) also formed coherent item clusters. Only two items (m10 and m13) exhibited minor cross-loadings (.33 and .31), which are within acceptable limits (Tabachnick & Fidell, 2019). Communalities (h²) ranged from 0.32 to 0.74; while some were lower (m16, m19), they were not low enough to warrant removal.

Table S9. Factor correlations (phi matrix).

|  | F1 | F2 | F3 | F4 |
| --- | --- | --- | --- | --- |
| F1 | 1.000 | 0.040 | 0.147 | 0.233 |
| F2 |  | 1.000 | 0.076 | 0.141 |
| F3 |  |  | 1.000 | 0.131 |
| F4 |  |  |  | 1.000 |
| **Note.** Inter-factor correlations ranged from 0.04 to 0.23, indicating weak associations and supporting discriminant validity. | | | | |

Table S9 shows inter-factor correlations ranging from 0.04 to 0.23. These low correlations provide strong evidence for discriminant validity, indicating that each factor is conceptually distinct (Brown, 2015). This finding also bodes well for the CFA, as low factor covariance suggests minimal measurement error and shared variance.

Table S10. Total variance explained after item removal (m1, m2, m4).

| Factor | SS Loadings | Proportion of Variance | Cumulative Variance (%) |
| --- | --- | --- | --- |
| F1 | 2.708 | 0.159 | 15.90 |
| F2 | 1.974 | 0.116 | 27.50 |
| F3 | 1.897 | 0.112 | 38.70 |
| F4 | 1.918 | 0.113 | 50.00 |

As shown in Table S10, the four factors now explain 50.0% of the total variance, meeting the 50% threshold (Hair et al., 2019). The variance is well-distributed (15.9%, 11.6%, 11.2%, 11.3%). The removal of the low-loading items (m1, m2, m4) resulted in a significant increase in explained variance (from 44.3% to 50.0%), indicating a more coherent and statistically robust structure.

**Parallel Analysis**

Table S11. Results of Horn’s parallel analysis.

| Component | Real Eigenvalue | Random Eigenvalue (95th Percentile) |
| --- | --- | --- |
| 1 | 3.96 | 1.59 |
| 2 | 2.14 | 1.47 |
| 3 | 1.68 | 1.39 |
| 4 | 1.39 | 1.33 |
| 5 | 1.18 | 1.27 |
| 6 | 1.13 | 1.21 |
| 7 | 0.95 | 1.17 |
| 8 | 0.93 | 1.12 |
| 9 | 0.84 | 1.08 |
| 10 | 0.75 | 1.03 |
| Note. Real eigenvalues exceeding the 95th percentile of random eigenvalues indicate the number of factors to retain. Based on this criterion, **four factors** were retained for subsequent exploratory analysis. | | |

Horn’s (1965) Parallel Analysis was used to determine the number of latent factors. The analysis compared the eigenvalues from the actual data (N=285, p=20) against the 95th percentile eigenvalues from 1000 random simulations. As shown in Table S11 and Figure S1, the actual eigenvalues for the first four components (3.96, 2.14, 1.68, 1.39) exceeded their corresponding random eigenvalues (1.59, 1.47, 1.39, 1.33). This finding supports a four-factor structure for the EFA.

Figure S1. Results of Horn’s parallel analysis (suggested number of factors: 4).


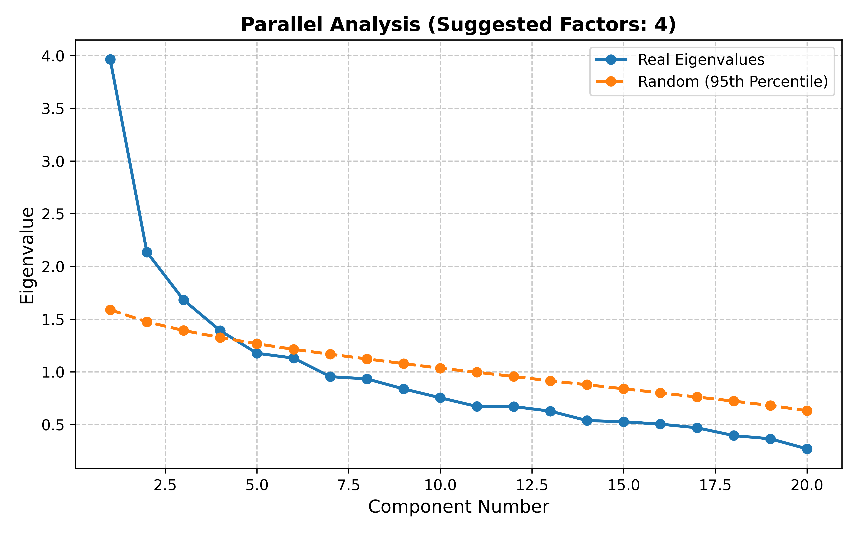


Figure S1 illustrates the Parallel Analysis results. The real eigenvalues (blue line) are compared against the 95th percentile random eigenvalues (orange dashed line). The first four components where the real eigenvalue exceeds the random eigenvalue statistically support the use of a four-factor solution in the EFA.

**FINAL EFA ANALYSIS**

**Exploratory Factor Analysis**

Table S12. Final pattern matrix and communalities after item exclusion (m1, m2, m4).

| Item | F1 | F2 | F3 | F4 | Communality |
| --- | --- | --- | --- | --- | --- |
| m3 | 0.51 |  |  |  | 0.47 |
| m5 |  |  | 0.86 |  | 0.73 |
| m6 |  | 0.74 |  |  | 0.56 |
| m7 |  | 0.78 |  |  | 0.62 |
| m8 |  | 0.61 |  |  | 0.44 |
| m9 |  |  | 0.85 |  | 0.73 |
| m10 |  |  | 0.53 |  | 0.42 |
| m11 | 0.62 |  |  |  | 0.42 |
| m12 | 0.82 |  |  |  | 0.67 |
| m13 | 0.66 |  |  |  | 0.52 |
| m14 | 0.59 |  |  |  | 0.44 |
| m15 | 0.73 |  |  |  | 0.54 |
| m17 |  |  |  | 0.64 | 0.48 |
| m18 |  |  |  | 0.72 | 0.53 |
| m20 |  |  |  | 0.52 | 0.44 |
| Note. Only factor loadings ≥ 0.30 are reported. Extraction method: Principal Axis Factoring (PAF); rotation: Direct Oblimin. | | | | | |

The final pattern matrix (Table S12) confirms a meaningful and interpretable four-factor solution. All item loadings were strong, ranging from 0.51 to 0.86. Factor 1 (m3, m11-m15) remained the strongest dimension (.51–.82). Factors 2 (m6-m8) and 3 (m5, m9, m10) were structurally consistent (.61–.86). Factor 4 (m17, m18, m20) showed moderate loadings (.52–.72). Minor cross-loadings for m3 (.37) and m10 (.34) were within acceptable limits (Tabachnick & Fidell, 2019). Communalities were strong (.42 to .73), indicating all items were well-explained by the factors.

Table S13. Factor correlations (Phi matrix).

|  | F1 | F2 | F3 | F4 |
| --- | --- | --- | --- | --- |
| F1 | 1.00 | 0.06 | 0.13 | 0.16 |
| F2 |  | 1.00 | .067 | 0.10 |
| F3 |  |  | 1.00 | 0.09 |
| F4 |  |  |  | 1.00 |

Table S13 shows very low inter-factor correlations (r = .06–.16), providing strong evidence for the scale's discriminant validity. This suggests each factor represents a distinct conceptual component (Brown, 2015) and supports a clean multidimensional structure.

Table S14. Total variance explained.

| Factor | SS Loadings | Proportion of Variance | Cumulative Variance (%) |
| --- | --- | --- | --- |
| F1 | 2.74 | 0.18 | 18.30 |
| F2 | 1.97 | 0.13 | 31.50 |
| F3 | 1.85 | 0.12 | 43.80 |
| F4 | 1.46 | 0.09 | 53.50 |

As shown in Table S14, the four factors explained 53.5% of the total variance, exceeding the 50% threshold (Hair et al., 2019). The variance explained was 18.3%, 13.2%, 12.3%, and 9.7%, respectively. The increase in total variance explained (from 44.3% to 53.5%) after removing items with low loadings demonstrates a more parsimonious and coherent final factor structure. Post-hoc checks confirmed the data remained suitable (KMO > .70, Bartlett's *p* < .001).

### RASCH ANALYSIS

#### Rasch-1: Unidimensionality Assumption (5-point Likert)

In the advanced stages of scale development, each subscale of the four-factor structure identified by EFA was evaluated using the Rasch Rating Scale Model (RSM). A fundamental assumption of Rasch analysis is that each factor exhibits a unidimensional structure. This assumption was tested by examining the Principal Component Analysis (PCA) of standardized residuals. If a factor is unidimensional, the first eigenvalue of the unexplained variance (residuals) is expected to be below 2.0 (Linacre, 2020).

Table S15. Rasch Model PCA of Residuals Results for Unidimensionality Assumption

| Factor | Item | Residual PCA 1st Eigenvalue |
| --- | --- | --- |
| F1 | m1-m6 | 1.47 |
| F2 | m7-m9 | - |
| F3 | m10-m12 | 1.89 |
| F4 | m13-m15 | 1.72 |

Table S15 shows the unidimensionality test for each factor. The assumption was supported for Factor 1 (m1–m6), Factor 3 (m10–m12), and Factor 4 (m13–m15), with first residual eigenvalues of 1.47, 1.89, and 1.72, respectively. However, Factor 2 (m7–m9) failed this test, with a residual eigenvalue of 2.30

#### Item Response Theory (Rasch) Analysis and Item Deletion:

**Category Structure Analysis (5-point Likert)**

After confirming unidimensionality for F1, F3, and F4, the next step was to evaluate the functioning of the Likert-type category structure. Using the RSM, the category thresholds for each factor were examined. This analysis tests whether the categories are "ordered," meaning participants use adjacent response options in a logical, incremental sequence of difficulty or endorsement. The results showed that for F1, F3, and F4, all category thresholds were properly ordered. This confirms that the 5-point Likert scale was interpreted by participants as intended. As no "disordered thresholds" were found, category collapsing was not deemed necessary *at this stage*, and analyses proceeded with the 5-point structure. (Note: F2 was excluded from this step as it failed the unidimensionality test).

**Item Fit (5-point Likert)**

With ordered categories confirmed, the fit of the items and category thresholds to the model was examined using Infit and Outfit Mean-Square (MNSQ) statistics. MNSQ values between 0.5–1.5 are considered acceptable, values > 1.5 indicate "noise" (misfit), and values < 0.5 indicate "overfit" (Linacre, 2020). ZSTD (t-values) were considered only if exceptionally high, with MNSQ being the primary criterion.

Table S16. Rasch Model Item and Category Fit Results (Infit/Outfit MNSQ)

| Factor | Item | Infit MNSQ | Infit t | Outfit MNSQ | Outfit t |
| --- | --- | --- | --- | --- | --- |
| F1 | m1 | 1.170 | 1.686 | 1.207 | 2.008 |
| (m1-m6) | m2 | 1.203 | 1.853 | 1.074 | 0.684 |
|  | m3 | 1.006 | 0.081 | 0.797 | -1.960 |
|  | m4 | 0.990 | -0.075 | 0.981 | -0.202 |
|  | m5 | 0.954 | -0.423 | 1.000 | -0.025 |
|  | m6 | 1.264 | 2.062 | 0.831 | -1.500 |
|  | Cat1 | 1.542 | >20 | 1.323 | >12 |
|  | Cat2 | 1.695 | >27 | 1.410 | >17 |
|  | Cat3 | 1.844 | >33 | 1.491 | >21 |
| F3 | m10 | 0.972 | -0.401 | 0.917 | -1.240 |
| (m10-m12) | m11 | 0.867 | -2.025 | 0.857 | -2.195 |
|  | m12 | 1.256 | 3.270 | 1.265 | 3.360 |
|  | Cat1 | 1.521 | >16 | 1.518 | >16 |
|  | Cat2 | 1.488 | >23 | 1.455 | >22 |
|  | Cat3 | 1.430 | >24 | 1.379 | >21 |
| F4 | m13 | 1.119 | 1.247 | 1.027 | 0.300 |
| (m13-m15) | m14 | 1.054 | 0.831 | 1.069 | 1.058 |
|  | m15 | 0.946 | -0.844 | 0.957 | -0.675 |
|  | Cat1 | 1.487 | >18 | 1.478 | >18 |
|  | Cat2 | 1.411 | >23 | 1.405 | >23 |
|  | Cat3 | 1.434 | >20 | 1.424 | >19 |
| Note: F2 was not included in the analysis due to a technical convergence error. Critical values (t > 2.0 or MNSQ > 1.5) are indicated in bold. Since category t-values were excessively high, only MNSQ values were interpreted. | | | | | |

Table S16 reveals that while item fit was generally acceptable for F1 and F4 (with m12 in F3 showing borderline misfit), the primary source of misfit was the category thresholds, not the items themselves. Specifically, Category 1 (Cat1) in Factor 1 and Category 1 (Cat1) in Factor 3 showed significant misfit (MNSQ > 1.5). This finding indicated that the 5-point Likert scale did not fully meet the functional expectations of the Rasch model and that response options were being used inconsistently. Therefore, to improve the psychometric quality, category collapsing was recommended. The analyses were re-run with responses re-coded into three categories (e.g., 1–2 / 3 / 4–5).

#### Rasch-2: Unidimensionality (3-point Likert)

The previous analysis identified significant category misfit (Infit MNSQ > 1.5) in the 5-point Likert structure. To address this, all items were re-coded into a 3-category format (e.g., 1–2 = "Low," 3 = "Medium," 4–5 = "High"). The unidimensionality assumption was then re-tested on this new 3-category data via Residual PCA (target eigenvalue < 2.0; Linacre, 2020).

Table S17. Unidimensionality Test Results After Category Collapsing (3-point Scale) (N=477)

| Factor | Item | Category Structure | Residual PCA 1st Eigenvalue |
| --- | --- | --- | --- |
| F1 | m1-m6 | 3-point | 1.497 |
| F2 | m7-m9 | 3-point | 1.585 |
| F3 | m10-m12 | 3-point | 1.832 |
| F4 | m13-m15 | 3-point | 1.803 |
| Note: A Residual PCA 1st Eigenvalue > 2.0 indicates a violation of the unidimensionality assumption (Linacre, 2020). | | | |

Table S17 shows that after re-coding, all four factors met the unidimensionality assumption (all eigenvalues < 2.0). Notably, the technical convergence error previously seen for F2 was resolved. This finding confirms that collapsing the categories significantly improved the stability of the measurement model.

**Category Structure Analysis (3-point Likert)**

The re-analysis of category thresholds (Threshold Analysis) on the 3-point data confirmed that the "category misfit" issue observed on the 5-point scale was resolved. The 3-point structure (Low/Medium/High) was found to be ordered and functionally sound for all four factors. This indicates that participants perceived the collapsed categories logically and consistently.

**Item Fit (3-point Likert)**

Item fit analysis was re-run on the 3-point scale data. Misfit was defined as MNSQ > 1.5 and/or *t* > 2.0.

Table S18. Rasch Model Item Fit Statistics on Re-coded (3-point Scale) Data (N=477)

| Factor | Item | Infit MNSQ | Infit t | Outfit MNSQ | Outfit t |
| --- | --- | --- | --- | --- | --- |
| F1 | m1 | 1.104 | 0.754 | 1.118 | 0.523 |
| (m1-m6) | m2 | 1.075 | 0.491 | 0.908 | -0.947 |
|  | m3 | 0.967 | -0.138 | 1.016 | -0.136 |
|  | m4 | 0.972 | -0.176 | 1.488 | 2.438 |
|  | m5 | 0.870 | -0.821 | 1.110 | 0.480 |
|  | m6 | 0.971 | -0.084 | 1.023 | -0.423 |
| F2 | m7 | 1.009 | 0.154 | 0.998 | -0.043 |
| (m7-m9) | m8 | 0.969 | -0.428 | 0.932 | -1.049 |
|  | m9 | 1.039 | 0.615 | 1.070 | 1.033 |
| F3 | m10 | 0.938 | -0.834 | 0.861 | -2.059 |
| (m10-m12) | m11 | 0.861 | -1.963 | 0.806 | -2.932 |
|  | m12 | 1.224 | 2.426 | 1.530 | 5.076 |
| F4 | m13 | 1.184 | 0.944 | 1.099 | 0.475 |
| (m13-m15) | m14 | 1.056 | 0.906 | 1.033 | 0.530 |
|  | m15 | 0.919 | -1.227 | 0.965 | -0.563 |
| **Note: Factor 2 was successfully included in the analysis on the 3-point scale. Critical misfit values (MNSQ > 1.5 and t ≥ 2.0) are indicated in bold.*** | | | | | |

The results in Table S18 were pivotal. All items within F1, F2, and F4 demonstrated excellent fit within the ideal range. The only misfit was found in F3. Item m12 showed significant misfit, with both its MNSQ (1.530) and *t*-statistic (5.076) exceeding the critical thresholds. This finding indicated that m12 was introducing "noise" and weakening the overall factor structure. Therefore, a decision was made to remove item m12 from the scale.

#### Differential Item Functioning (DIF)

Following the removal of m12 (which also necessitated the removal of the now-unstable Factor 3), the remaining items in F1, F2, and F4 were tested for Differential Item Functioning (DIF) by Gender (Female/Male).

Table S19. DIF Analysis Wald Test Results (Group: Gender)

| Factor | Item | Bonferroni Criterion (α) | Lowest (Critical) p-value |
| --- | --- | --- | --- |
| F1 | m1 | 0.008 | 0.36 |
| (m1-m6) | m2 |  | 0.83 |
|  | m3 |  | 0.43 |
|  | m4 |  | 0.36 |
|  | m5 |  | 0.86 |
|  | m6 |  | 0.51 |
| F2 | m7 | 0.016 | 0.53 |
| (m7-m9) | m8 |  | 0.37 |
|  | m9 |  | 0.70 |
| F4 | m13 | 0.016 | 0.21 |
| (m13-m15) | m14 |  | 0.23 |
|  | m15 |  | 0.05 |
| Note: The "Lowest p-value" in the table indicates the smallest p-value among the threshold p-values belonging to each item. | | | |

As shown in Table S19, no significant DIF by gender was detected for any item. The lowest *p*-value observed (for m15, *p* = .05) was well above the Bonferroni-corrected critical threshold for its factor (α = 0.0167). This indicates that the scale functions fairly for both women and men, with no evidence of measurement bias.

### Model Refinement and CFA Sequence

#### Determining the 3-Factor Model (Based on Rasch)

The Rasch analyses provided a clear path for methodological refinement:

1. **Category Structure:** The 5-point scale showed significant category misfit (MNSQ > 1.5). This was resolved by collapsing the scale into 3 categories (Low/Medium/High).
2. **Item Fit:** On the 3-point scale, item m12 showed significant misfit (MNSQ = 1.530, *t* = 5.076) and was removed.
3. **Factor Removal:** The removal of m12 left F3 with only two items, rendering it structurally weak. Therefore, Factor 3 (m10, m11, m12) was removed entirely.
4. **DIF:** The remaining items showed no gender DIF.

This iterative process resulted in a psychometrically stronger 12-item, 3-factor model (F1, F2, F4) to be tested via CFA.

Table S20. Final Model Structure After Rasch Analysis (12 Items, 3 Factors)

| Final Factor | Included Item (Total 12) |
| --- | --- |
| F1 | m1, m2, m3, m4, m5, m6 |
| F2 | m7, m8, m9 |
| F4 | m13, m14, m15 |
| (Excluded Factor) | (F3: m10, m11, m12) |

#### CFA of the 3-Factor Model (F1, F2, F4)

The 12-item, 3-factor model (F1, F2, F4) identified by Rasch was tested using CFA with the WLSMV estimator on the 3-point ordinal data.

Table S21. CFA Fit Indices for the 3-Factor Model (12 Item) (N = 477)

| Model Fit Index | Observed Value | Acceptable Threshold (Hu & Bentler, 1999) |
| --- | --- | --- |
| X^2^/df | 1.35 (69.03 / 51.00) | < 3.0 |
| p-value (Chi-Square) | 0.04 | > .05 |
| CFI | 0.99 | > .95 |
| TLI | 0.98 | > .95 |
| RMSEA | 0.02 | < .06 |
| SRMR | 0.08 | < .08 |

The fit indices for this 3-factor model were mixed. While CFI (.991), TLI (.988), and RMSEA (.027) indicated excellent fit, the SRMR (.085) was slightly above the .08 threshold, suggesting some residual variance.

Table S22. Standardized Factor Loadings and Inter-Factor Correlations for the 3-Factor Model

| Factor | Item | Standardized Loading (std.all) |
| --- | --- | --- |
| F1 | m1 | 0.74 |
|  | m2 | 0.84 |
|  | m3 | 0.74 |
|  | m4 | 0.80 |
|  | m5 | 0.8 |
|  | m6 | 0.86 |
| F2 | m7 | 0.70 |
|  | m8 | 0.92 |
|  | m9 | 0.67 |
| F4 | m13 | 0.59 |
|  | m14 | 0.41 |
|  | m15 | 0.53 |
| Factor Correlations |  | Standardized Correlation (r) |
| F1 ~~ F2 |  | 0.44 |
| F1 ~~ F4 |  | 0.84 |
| F2 ~~ F4 |  | 0.77 |
| Note: All factor loadings and correlations are statistically significant (p < .001). The 'est' column in the output indicates unstandardized loadings, while the 'std.all' column indicates standardized loadings. | | |

Table S22 revealed two critical structural problems:

1. **Weak Loading:** Item m14 had a very weak, unacceptable loading on F4 (λ = .409).
2. **Lack of Discriminant Validity:** The correlation between F1 and F4 was excessively high (r = .840), indicating severe multicollinearity. These two factors were not measuring distinct constructs.

**Conclusion:** Although some fit indices were good, this 3-factor model was rejected as structurally invalid due to the extremely high correlation between F1 and F4.

#### Rasch-3 and CFA: Testing the Final 2-Factor Model

**Unidimensionality (F1+F4, F2)** The CFA finding (r = .840) strongly suggested that F1 and F4 were not separate, empirically supporting the hypothesis that they should be merged into a single, unified factor. To test this new "Dual Architecture" hypothesis, we returned to the Rasch analysis.

**We created two new factors:** Factor 1 (Merged) (9 items: m1-m6 + m13-m15) and Factor 2 (3 items: m7-m9). Both were tested for unidimensionality on the 3-point data.

Table S23. Unidimensionality Test Results for the Alternative 2-Factor Model

| New Factor Structure | Item | Residual PCA 1st Eigenvalue |
| --- | --- | --- |
| F1 (Merged) | m1-m6, m13-m15 (9 Item) | 1.55 |
| F2 | m7-m9 (3 Item) | 1.59 |
| Note: A Residual PCA first Eigenvalue less than 2.0 is suggestive of unidimensionality (Linacre, 2020). Results from the Rasch unidimensionality test for the redefined two-factor structure are shown in Table S23. | | |

As shown in Table S23, the analysis strongly supported this theoretical shift. Both the Merged F1 (eigenvalue = 1.55) and F2 (eigenvalue = 1.59) passed the unidimensionality test (value < 2.0).

Item Fit (2-Factor Model) Item fit was then assessed for this new 2-factor structure.

Table S24. Rasch Item Fit Statistics for the 2-Factor Model

| Final Factor | Item | Infit MNSQ | Infit t | Outfit MNSQ | Outfit t |
| --- | --- | --- | --- | --- | --- |
| F1 | m1 | 1.078 | 0.581 | 0.988 | -0.120 |
| (Merged F1+F4) | m2 | 1.122 | 0.780 | 0.968 | -0.278 |
| (9 Item) | m3 | 0.967 | -0.138 | 0.896 | -0.641 |
|  | m4 | 0.868 | -0.949 | 0.792 | -1.614 |
|  | m5 | 0.873 | -0.795 | 0.833 | -1.121 |
|  | m6 | 0.970 | -0.088 | 0.690 | -1.714 |
|  | m13 | 1.076 | 0.431 | 1.365 | 1.616 |
|  | m14 | 1.129 | 1.895 | 1.221 | 3.083 |
|  | m15 | 0.996 | -0.054 | 1.168 | 2.203 |
|  | Cat1 (Threshold) | 1.404 | 7.655 | 1.218 | 4.323 |
| F2 | m7 | 1.000 | -0.002 | 0.970 | -0.545 |
| (m7-m9) | m8 | 0.980 | -0.259 | 0.984 | -0.315 |
| (3 Item) | m9 | 1.020 | 0.317 | 1.009 | 0.081 |
|  | Cat1 (Threshold) | 1.282 | 5.686 | 1.232 | 4.738 |
| *Note: Critical values (MNSQ > 1.5 or t > 2.0) are indicated in bold. | | | | | |

As shown in Table S24, all 9 items in the Merged F1 factor and all 3 items in F2 were within the ideal MNSQ range (0.5–1.5). Although items m14 and m15 had *t*-values slightly above 2.0, their MNSQ fit was good, indicating this was not a critical issue.

**DIF (2-Factor Model)** Finally, the 12 items (in their 2-factor structure) were tested for gender DIF.

Table S25. DIF Analysis Results for the 2-Factor Model (Group: Gender)

| Final Factor | Number of Items | Bonferroni Criterion (α) | Lowest (Critical) p-value |
| --- | --- | --- | --- |
| F1 (Merged F1+F4) | 9 Item | 0.0056 | 0.043 (item m15) |
| F2 (m7-m9) | 3 Item | 0.0167 | 0.368 (item m8) |
| Note: The p-values were obtained from the Wald Test results in the R output. | | | |

As shown in Table S25, no gender DIF was found. The lowest *p*-value (*p* = .043 for m15) was well above the Bonferroni-corrected threshold (α = 0.0056). This confirmed the 12-item, 2-factor model was fair for gender comparisons.

Table S26. Final Model Structure (12 Items, 2 Factors)

| Final Factor | Included Items (Total 12) |
| --- | --- |
| Digital Fidelity Ethics F1 (New) | m1, m2, m3, m4, m5, m6, m13, m14, m15 |
| Boundary Management F2 (New) | m7, m8, m9 |
| (Excluded Factor) | (3: m10, m11, m12) |

The comprehensive psychometric refinement process (CFA and Rasch) resulted in a final, theoretically robust 2-factor, 12-item model (Factor 1: "Digital Fidelity Ethics," 9 items; Factor 2: "Boundary Management," 3 items).

#### CFA of the 2-Factor (12-Item) Model

This final 12-item, 2-factor structure was tested using CFA (WLSMV estimator).

Table S27. CFA Fit Indices for the 2-Factor Model (12 Items) (N=477)

| Model Fit Index | Observed Value | Acceptable Threshold (Hu & Bentler, 1999) |
| --- | --- | --- |
| X^2^/df | 2.38 (126.272 / 53.000) | < 3.0 |
| p-value | 0.000 | > .05 |
| CFI | 0.928 | > .90 |
| TLI | 0.910 | > .90 |
| RMSEA | 0.054 | < .06 |
| SRMR | 0.096 | < .08 |

The 12-item model showed acceptable, but not excellent, fit. While χ²/df (2.38) and RMSEA (.054) were good, the CFI (.928) and TLI (.910) were below the .95 threshold, and the SRMR (.096) was unacceptably high.

Table S28. Standardized Factor Loadings for the 2-Factor (12-Item) Model

| Factor | Item | Standardized Factor Loading (std.all) |
| --- | --- | --- |
| Digital Fidelity Ethics F1 (New) | m1 | 0.725 |
| (9 items) | m2 | 0.823 |
|  | m3 | 0.724 |
|  | m4 | 0.778 |
|  | m5 | 0.788 |
|  | m6 | 0.860 |
|  | m13 | 0.582 |
|  | m14 | 0.407 |
|  | m15 | 0.521 |
| Boundary Management F2 (New) | m7 | 0.695 |
| (3 items) | m8 | 0.928 |
|  | m9 | 0.665 |
| Factor Correlation |  | Standardized Correlation (r) |
| Digital Fidelity Ethics ~~ Boundary Management |  | 0.536 |
| Note: All factor loadings and correlations are statistically significant (p < .001). | | |

Table S28 revealed the source of the misfit. While discriminant validity was excellent (r = .536), the loading for m14 was unacceptably weak (λ = .407). This item was clearly not measuring the same construct as the other 8 items in F1 and was harming the model's structural integrity.

#### CFA of the 11-Item Model (m14 removed)

To resolve this, m14 was removed, and an 11-item model (F1: 8 items; F2: 3 items) was tested.

Table S29. Model Fit Comparison After Removing m14 (11-Item Model)

| Model Fit Index | Previous Model (12 Items) | Revised Model (11 Items) |
| --- | --- | --- |
| CFI | 0.928 | 0.929 |
| TLI | 0.910 | 0.910 |
| RMSEA | 0.054 | 0.058 |
| SRMR | 0.096 | 0.095 |

As Table S29 shows, removing m14 did not solve the problem. The fit indices (CFI/TLI) showed no improvement, and the SRMR remained unacceptably high (.095).

Table S30. Standardized Factor Loadings for the 11-Item Model

| Factor | Item | Standardized Factor Loading (std.all) |
| --- | --- | --- |
| Digital Fidelity Ethics F1 (New) | m1 | 0.720 |
| (8 Items) | m2 | 0.840 |
|  | m3 | 0.740 |
|  | m4 | 0.776 |
|  | m5 | 0.790 |
|  | m6 | 0.863 |
|  | m13 | 0.580 |
|  | m15 | 0.511 |
| Boundary Management F2 (New) | m7 | 0.698 |
| (3 Items) | m8 | 0.922 |
|  | m9 | 0.668 |
| Factor Correlation |  | Standardized Correlation (r) |
| Digital Fidelity Ethics ~~ Boundary Management |  | 0.517 |
| Note: All factor loadings and correlations are statistically significant (p < .001). | | |

Table S30 revealed why the fit did not improve. With m14 removed, m15 was now the weakest item, showing a borderline, weak loading (λ = .511). This indicated that the misfit was not isolated to m14, and m15 was also a poorly performing item. This led to the final CFA (presented in the main text, Table 2) where *both* m14 and m15 were removed, resulting in the final, well-fitting 10-item model.

### Rasch Targeting and Validation Scale Psychometrics

#### Targeting (Final 10-Item Model)

Rasch targeting analysis was conducted to assess the match between the item difficulty and the sample's ability (trait level). An ideal match is a difference (mismatch) of 0.0 logits, with |1.0| as an acceptable limit.

Table S31. Item-Person Targeting Statistics for the Final 10-Item, 2-Factor Model

| Final Factor | Number of Items | Mean Item Difficulty (Logit) | Mean Person Ability (Logit) | Targeting Difference (Mismatch) |
| --- | --- | --- | --- | --- |
| Digital Fidelity Ethics (F1) | 7 | -2.859 | -0.806 | +2.053 |
| Boundary Management (F2) | 3 | -0.494 | 0.161 | +0.655 |
| Note: Targeting Difference = Mean Person Ability – Mean Item Difficulty. | | | | |

As shown in Table S31, F2 (Boundary Management) showed good targeting (+0.655 logit), indicating its items were well-matched to the sample's ability level. However, F1 (Digital Fidelity Ethics) showed severe mistargeting (+2.053 logit). This large mismatch indicates that the F1 items were far too easy for the sample, confirming the "ceiling effect" discussed in the main text and explaining the low PSI reliability (the scale could not differentiate between high-scoring individuals).

#### Psychometric Properties of Validation Scales

**Relationship Assessment Scale (RAS)**

Table S32. Standardized Factor Loadings (λ) and Explained Variance (R²) for the RAS (Final 5-Item Model)

| Item | Factor Loading (λ) | Std. Error (SE) | z-value | p | Explained Variance (R²) |
| --- | --- | --- | --- | --- | --- |
| k1 | .908 | 0.012 | 83.637 | <.001 | .824 |
| k2 | .946 | 0.012 | 83.637 | <.001 | .895 |
| k3 | .942 | 0.011 | 91.339 | <.001 | .887 |
| k5 | .930 | 0.011 | 91.230 | <.001 | .866 |
| k6 | .898 | 0.014 | 71.703 | <.001 | .806 |
| Note. Item k1 was set as the reference. All factor loadings are significant at the *p* < .001 level. N=477. | | | | | |

As shown in Table S32, all 5 items of the final RAS model had exceptionally high and significant factor loadings (λ range = .898–.946, *p* < .001). The R² values (.806–.895) indicate that the single factor explains the vast majority of the variance in each item, supporting a robust, unidimensional structure.

Table S33. Reliability and Item Statistics for the 5-Item RAS Model

| Item | Corrected Item-Total Correlation (r_drop_) | Cronbach's α if Item Deleted | Mean | Standard Deviation (SD) |
| --- | --- | --- | --- | --- |
| k1 | .835 | .934 | 5.12 | 2.13 |
| k2 | .879 | .926 | 5.22 | 2.10 |
| k3 | .872 | .927 | 5.29 | 2.03 |
| k5 | .866 | .928 | 4.87 | 2.05 |
| k6 | .790 | .941 | 5.72 | 2.00 |
| Total Score (5 Items) |  | Cronbach's alpha = .944 |  |  |
| Note. N=477. r_drop_ = Corrected item-total correlation. Total scale McDonald’s Omega = .945 | | | | |

Table S33 confirms the scale's excellent internal consistency. Corrected item-total correlations (r_drop_) were very high (.790–.879). Both Cronbach’s Alpha (α = .944) and McDonald’s Omega (ω = .945) were well above the .90 threshold for "excellent" reliability.

**Attachment Styles Questionnaire (ASQ)**

Table S34. Standardized Factor Loadings (λ) and Explained Variance (R²) for the ASQ (Final 10-Item Model)

| Factor | Item | Factor Loading (λ) | Explained Variance (R²) |
| --- | --- | --- | --- |
| Secure Attachment (F1) | n1 | .548 | .301 |
|  | n2 | .907 | .823 |
|  | n3 | .759 | .576 |
|  | n4 | .537 | .288 |
| Anxious Attachment (F2) | n6 | .669 | .447 |
|  | n7 | .673 | .453 |
| Avoidant Attachment (F3) | n9 | .606 | .368 |
|  | n10 | .755 | .570 |
|  | n11 | .852 | .726 |
|  | n12 | .746 | .557 |
| Note: All factor loadings are significant at the p < .001 level. N=477. | | | |

Table S34 shows that all items in the 10-item ASQ model are statistically significant (*p* < .001), with factor loadings ranging from .537 to .907. This strong range supports the robustness of the measurement model and its three-dimensional structure.

Table S35. Inter-Factor Standardized Correlations for the ASQ Model

| Factor | 1 | 2 | 3 |
| --- | --- | --- | --- |
| 1. Secure Attachment (F1) | — |  |  |
| 2. Anxious Attachment (F2) | -.257** | — |  |
| 3. Avoidant Attachment (F3) | -.377** | .723** | — |
| Note. All correlations are significant at the p < .001 level. **p < .001 | | | |

Table S35 shows moderate correlations between the three factors (*p* < .001). The highest correlation was between F2 (Anxious) and F3 (Avoidant) (r = .723), which is below the threshold for discriminant validity concerns.

**Perceived Partner's Intentions Towards Infidelity Scale (P-ITIS)**

Table S36. Standardized Factor Loadings (λ) and Explained Variance (R²) for the P-ITIS (Single-Factor Model)

| Item | Factor Loading (λ) | Explained Variance (R²) |
| --- | --- | --- |
| h1 | .947 | .897 |
| h2 | .936 | .877 |
| h3 | .803 | .645 |
| h4 | .929 | .863 |
| h5 | .918 | .843 |
| Note: All factor loadings are significant at the p < .001 level. N=477. | | |

Table S36 demonstrates that all five items of the P-ITIS significantly represent a single latent factor (*p* < .001). Factor loadings were "excellent" (.803–.947), and the R² values (.645–.897) were very high. These findings support a robust, unidimensional structure with strong convergent validity.
